# A Covariant Non-Local Model of Bohm’s Quantum Potential

**DOI:** 10.3390/e25060915

**Published:** 2023-06-09

**Authors:** Roberto Mauri, Massimiliano Giona

**Affiliations:** 1 DICI, Department of Civil and Industrial Engineering, Università di Pisa, 56122 Pisa, Italy; 2 DICMA, Department of Industrial Engineering, Università di Roma La Sapienza, 00184 Roma, Italy; massimiliano.giona@uniroma.it

**Keywords:** non-local quantum mechanics, Bohm quantum potential, Madelung equation

## Abstract

Assuming that the energy of a gas depends non-locally on the logarithm of its mass density, the body force in the resulting equation of motion consists of the sum of density gradient terms. Truncating this series after the second term, Bohm’s quantum potential and the Madelung equation are obtained, showing explicitly that some of the hypotheses that led to the formulation of quantum mechanics do admit a classical interpretation based on non-locality. Here, we generalize this approach imposing a finite speed of propagation of any perturbation, thus determining a covariant formulation of the Madelung equation.

## 1. Introduction

In a recent article [1], we have shown that in a perfect fluid the Bohm potential can be obtained without any quantum mechanical prior assumption. In fact, unlike previous works (see the comprehensive review of Dürr et al. [2]) where the Madelung equation is derived starting from the Schrödinger equation, here the same result is obtained assuming a classical behavior, with a non-local logarithmic dependence of the free energy from the mass (or number) density. Let us start by reviewing this earlier work.

Consider the Hamilton–Jacobi equation for a particle of unit mass m = 1 located at position r and time *t* in a potential energy field, $V_{S}\left(r,t\right)$,

(1) $$\frac{\partial S}{\partial t}=-H\left(r,\nabla S,t\right);\;H\left(r,p,t\right)=\frac{1}{2}p^{2}+V_{S},$$

where *S* is the action, defined as the time integral of the Lagrangian function. Here, *S* depends on position r, time *t*, and momentum p, i.e., $S=S\left(r,t,p\right)$, where p=∇S, showing that the momentum (and the velocity v=p/m as well) can be expressed as the gradient of a scalar function. Note that by taking the gradient of Equation (1) we obtain Newton’s equation of motion:

(2) $$\frac{dp}{dt}=F_{S}=-\nabla V_{S},\;\mathrm{with}\;\frac{d}{dt}=\frac{\partial }{\partial t}+v\cdot \nabla$$

denoting the material derivative.

Now, let us interpret the action *S* as the velocity potential of an inviscid fluid (in fact, its momentum is p=∇S), assuming that this particle is one of the very many identical particles, not interacting with each other, that constitute an ideal fluid in isothermal conditions. Then, the governing equations, expressing the conservation of mass and momentum, read [3]

(3) $$\begin{array}{ccc} \frac{\partial \rho }{\partial t}+\nabla \cdot \left(\rho v\right)=0,\; & i.e., & \;\frac{d\rho }{dt}=-\rho \left(\nabla \cdot v\right), \end{array}$$

(4) $$\begin{array}{ccc} \frac{\partial \left(\rho v\right)}{\partial t}+\nabla \cdot \left(\rho vv\right)=\rho F_{S},\; & \;i.e., & \;\rho \frac{dv}{dt}=\rho F_{S}, \end{array}$$

where ρ is the mass density, while $F_{S}$ is the force exerted on a particle, defined in Equation (2). Note that ρ denotes both the mass density and the number density since, without loss of generality, we have assumed that particles have unit mass.

It should be stressed that ρ and v are mean values, defined within an elementary point volume, assuming local equilibrium. From a different point of view, we are neglecting fluctuations, i.e., the Ginzburg criterion is satisfied, so the mean field approximation can be applied (see Landau et al. [4]).

Classically, in Equation (4), the force $F_{S}$, denoted as $F_{S,cl}$, is often expressed as the sum of a pressure term plus an external conservative force, i.e.,

(5) $$F_{S,cl}=-\frac{1}{\rho }\nabla P-\nabla V,$$

where *P* is the pressure which, for an ideal fluid, is readily written as P=kTρ, so that

(6) $$F_{S,cl}=-\nabla V_{cl},\;\mathrm{where}\;V_{cl}=kT\ln\rho +V.$$

This expression, showing that the classical potential energy is the sum of a pressure-dependent inner part and an external part, assumes that the gas evolves under isothermal conditions. If we remove this assumption, an energy conservation equation should be added to Equations (3) and (4), as will be shown in future works.

Now, define a wave-like complex function, ψ,

(7) $$\psi =\sqrt{\rho }e^{iS/\hbar },$$

where $i=\sqrt{-1}$, ℏ=h/2π, and *h* is the Planck constant, imposing that both its real and imaginary components satisfy the Schrödinger equation,

(8) $$i\hbar \frac{\partial \psi }{\partial t}=-\frac{\hbar^{2}}{2}\nabla^{2}\psi +V_{cl}\psi ,$$

with $V_{cl}$ denoting the classical potential energy (6) acting upon the particle. The real part of Equation (8) coincides with Equation (3), showing that ρ can be interpreted as the probability to find a particle at location r and at time *t*. In addition, the imaginary part of Equation (8) yields the Hamilton–Jacobi Equation (1), where

(9) $$V_{S}=V_{cl}+V_{Q},\;\mathrm{with}\;V_{Q}=-\frac{\hbar^{2}}{2}\frac{\nabla^{2}\sqrt{\rho }}{\sqrt{\rho }}.$$

$V_{Q}$ is the Bohm potential [5,6] that is a central concept of the de Broglie–Bohm formulation of quantum mechanics (see reviews in [2,7]). Therefore, we conclude that the classical Hamilton–Jacobi Equation (1) is equivalent to the Schrödinger Equation (6), provided that $V_{S}=V_{cl}+V_{Q}$, that is, the potential energy is the sum of the classical potential energy and its quantum counterpart, i.e., the Bohm potential. To observe this, first consider that when the Hamiltonian *H* does not depend on time explicitly, then, applying separation of variables, we find that $S=W\left(r\right)-Et$, where *E* is the energy, which is constant when the system is isolated or at steady state. Here, *W* is called Hamilton’s characteristic function, and the quantum Hamilton–Jacobi equation becomes

(10) $$H\left(r,\nabla W\right)=E.$$

At steady state, momentum is nul, that is ∇S=0. Then, *W* is uniform (we may assume it to be zero, as it is determined within an arbitrary constant), so that S=−Et and therefore Equation (10) reduces to $V_{S}=E$. Then, considering Equation (9) and denoting $\phi \left(r\right)=\sqrt{\rho (r)}$, we find

(11) $$-\frac{\hbar^{2}}{2}\nabla^{2}\phi +V_{cl}\phi =E\phi ,$$

which coincides with the steady solution of the Schrödinger equation.

It should be observed that the Bohmian formulation, involving a second-order differential equation of motion in the presence of the quantum potential, reproduces the classical predictions of quantum mechanics only if the initial position $r\left(t=0\right)=r_{0}$ is distributed according to the Born rule, i.e., according to the density $\rho \left(r,0\right)$ and if the particle momentum is restricted to the value $p\left(t=0\right)=\nabla S\left(r,0\right)$ [8].

Finally, summarizing, from Equations (2) and (4), the force balance on each particle yields

(12) $$\frac{dp}{dt}=F_{S}=-\nabla V_{cl}-\nabla V_{Q}.$$

These results were obtained in 1927 by Madelung [9], showing that the Schrödinger equation for one-electron problems can be transformed into hydrodynamical equations of an ideal (i.e., non-viscous) gas in isothermal conditions, subjected to the action of both classical and quantum potentials.

## 2. The Quantum Potential

Although many researchers have stated that the Bohm quantum potential is due to non-local effects, there is no clear explanation of how that happens. Recently [1], recalling that, as lnρ is an additive integral of motion it must be proportional to the energy (see Landau et al. [4]), we propose the following non-local expression for the free energy per unit mass, $\hat{f}$:

(13) $$\hat{f}\left(r,t\right)=kT\int u\left(|r-r^{\prime }|\right)\;\ln\rho \left(r^{\prime },t\right)d^{3}r^{\prime }+V\left(r,t\right).$$

Here, $V\left(r,t\right)$ is the potential energy per unit mass resulting from the action of an external conservative force, while $u\left(x\right)$ is an interaction kernel between particles located at a distance $x=|r-r^{\prime }|$, with the normalization condition $\int u\left(x\right)d^{3}x=1$. Dropping for convenience the time dependence, expanding lnρ in Taylor series,

(14) $$\ln\rho \left(r+x\right)=\ln\rho \left(r\right)+x\cdot \nabla \ln\rho \left(r\right)+\frac{1}{2}xx:\nabla \nabla \ln\rho \left(r\right)+\cdots$$

and truncating the series after the second term (there can be no ∇lnρ term, due to the isotropy of the fluid), we find

(15) $$\hat{f}\left(r\right)={\hat{f}}_{th}\left(r\right)+\Delta {\hat{f}}_{nl}\left(r\right).$$

Here, the first term on the RHS is the usual classic (i.e., thermodynamic) free energy (per unit mass) of an ideal gas, coinciding with the classical potential energy (6),

(16) $${\hat{f}}_{th}\left(r\right)=kT\ln\rho \left(r\right)+V\left(r\right)$$

while

(17) $$\Delta {\hat{f}}_{nl}\left(r\right)=-\frac{1}{2}kTa^{2}\nabla^{2}\ln\rho \left(r\right)$$

is the non-local part, with

(18) $$a^{2}=-\int x^{2}u\left(x\right)d^{3}x$$

denoting the square of a characteristic length, *a*. Note the negative sign in Equation (18), revealing that particles are assumed to attract each other. Higher-order terms in the expansion (14) can be neglected under quite general conditions, as discussed in Sections 3 and 4 of Ref. [1]. In fact, assuming an exponentially decreasing interaction kernel u(x) in [1], we show that the additional terms, correcting the Madelung equation, are very small.

Now, define the free energy per unit volume, $\left(\rho \hat{f}\right)$, so that the total free energy is given by the following functional:

(19) $$F\left[\rho \left(r\right)\right]=\int_{V}\rho \left({\hat{f}}_{th}-\frac{1}{2}kTa^{2}\nabla^{2}\ln\rho \right)d^{3}r,$$

where *V* is the total volume, that we assume to be infinite. Integrating parts with the assumption that ρ→0 exponentially as r→∞, we find

(20) $$F\left[\rho \left(r\right)\right]=\int_{V}fd^{3}r;\;f=\rho \left[{\hat{f}}_{th}+\frac{1}{2}kTa^{2}{\left(\nabla \ln\rho \right)}^{2}\right],$$

where *f* denotes the effective free energy per unit volume.

Imposing that the free energy is minimal, under the constraint of mass conservation, i.e., $\int \rho d^{3}r=const$, we obtain the following generalized chemical potential:

(21) $$\mu =\frac{\delta f}{\delta \rho }=\frac{\partial f}{\partial \rho }-\nabla \cdot \frac{\partial f}{\partial \nabla \rho }=\mu_{th}+\mu_{nl}$$

where

(22) $$\mu_{th}=d\left(\rho {\hat{f}}_{th}\right)/d\rho ={\hat{f}}_{th}+kT,$$

and

(23) $$\mu_{nl}=-kTa^{2}\left[\nabla^{2}\ln\rho +\frac{1}{2}{\left(\nabla \ln\rho \right)}^{2}\right].$$

Here, $\mu_{th}$ is the thermodynamic chemical potential (an energy per unit mass) which, apart from an irrelevant additive constant (i.e., kT), coincides with the thermodynamic free energy (i.e., the classical potential energy), while $\mu_{nl}$ is the non-local contribution. After a straightforward calculation (see Ref. [1]), it can be shown that the non-local potential (23) can be expressed in the following equivalent form:

(24) $$\mu_{nl}=-2kTa^{2}\frac{\nabla^{2}\sqrt{\rho }}{\sqrt{\rho }}.$$

In particular, imposing that at leading order the non-local potential (24) reduces to Bohm’s quantum potential (9), i.e.,

(25) $$\mu_{nl}=V_{Q}=-\frac{\hbar^{2}}{2}\frac{\nabla^{2}\sqrt{\rho }}{\sqrt{\rho }},$$

then *a* coincides with the thermal de Broglie wavelength,

(26) $$a=\frac{\hbar }{2\sqrt{kT}}.$$

The equations of motion can be determined by applying a variational principle [10] to derive the Euler Equation (2) for a compressible, inhomogeneous fluid where $F_{S}$ is a body force, driven by density gradients in the fluid. The same result can be obtained more heuristically by applying Noether’s theorem [11] or by the least-action principle [12]. At the end, we find Equations (3) and (4), with the following force per unit mass:

(27) $$F_{S}=-\nabla \mu =-\nabla \mu_{th}-\nabla \mu_{nl}.$$

Let us consider the two terms on the RHS separately. On one hand, we have

(28) $$F_{th}=-\nabla \mu_{th},\;\mathrm{with}\;\mu_{th}=kT\ln\rho +V,$$

and therefore, considering that P=kTρ,

(29) $$\rho F_{th}=-\rho \nabla \mu_{th}=-\nabla P-\rho \nabla \phi .$$

On the other hand, from Equations (23) and (26), we define a non-local reversible body force, which is usually referred to as the Korteweg force [10]:

(30) $$F_{nl}=-\nabla \mu_{nl}=\frac{\hbar^{2}}{2}\nabla \left(\frac{\nabla^{2}\sqrt{\rho }}{\sqrt{\rho }}\right).$$

Finally, summarizing, from Equation (2), the momentum balance per unit mass yields the Madelung equation:

(31) $$\frac{\partial v}{\partial t}+v\cdot \nabla v=F_{S}=-\nabla V_{cl}-\nabla V_{Q}.$$

Equation (31) can also be written as a quantum Bernoulli equation as follows [13]:

(32) $$\frac{\partial v}{\partial t}=-\nabla E=-\nabla \left(\frac{1}{2}v^{2}+V_{cl}+V_{Q}\right),$$

where the RHS denotes the gradient of the total energy, *E*, that is the sum of kinetic energy, potential energy, and quantum potential energy.

It should be stressed that the Madelung Equation (31) (as well as the quantum Bernoulli Equation (32)) is a leading-order approximation of the more complete equation of motion where the full gradient expansion (14) is accounted for (see Equation (38) of Ref. [1]).

## 3. Retarded Potential and Covariant Formulation

The non-local constitutive Equation (13) implies that density changes in r’ determine an instantaneous variation of the free energy at r, with an infinite propagation velocity. To correct this obviously unrealistic assumption, Equation (13) is replaced with the following expression:

(33) $$\hat{f}\left(r,t\right)=kT\int u\left(|r-r^{\prime }|\right)\;\ln\rho \left(r^{\prime },t^{\prime }\right)d^{3}r^{\prime }+V\left(r,t\right),$$

where

(34) $$t^{\prime }=t-\frac{\left|r-r^{\prime }\right|}{c}$$

is a retarded time and *c* denotes the propagation velocity.

Expanding lnρ in the Taylor series,

(35) $$\begin{array}{c} \ln\rho \left(r+x,t+\frac{x}{c}\right)=\ln\rho \left(r,t\right)+x\cdot \nabla \ln\rho \left(r,t\right)+\frac{x}{c}\frac{\partial }{\partial t}\ln\rho \left(r,t\right) \end{array}$$

(36) $$\begin{array}{c} +\frac{1}{2}xx:\nabla \nabla \ln\rho \left(r\right)+\frac{1}{2}\frac{x^{2}}{c^{2}}\frac{\partial^{2}}{\partial t^{2}}\ln\rho \left(r,t\right)+\cdots \end{array}$$

and truncating the series after the quadratic term (the linear terms give no contribution due to simple spatial and temporal symmetry considerations), we find Equation (15), i.e., $\hat{f}={\hat{f}}_{th}+\Delta {\hat{f}}_{nl}$, where ${\hat{f}}_{th}$ is again the classical potential energy (16), while the non-local contribution reads

(37) $$\Delta {\hat{f}}_{nl}=\frac{1}{2}kTa^{2}\left(\frac{1}{c^{2}}\frac{\partial^{2}}{\partial t^{2}}-\nabla^{2}\right)\ln\rho \left(r,t\right)=\frac{1}{2}kTa^{2}□\ln\rho ,$$

with

(38) $$□=\frac{1}{c^{2}}\frac{\partial^{2}}{\partial t^{2}}-\nabla^{2}$$

denoting the d’Alambertian wave operator. Equation (37) is the Lorentz-invariant form of the non-local energy (17). This result has been obtained previously by Holland [14] or, more recently, by Nicolić [15,16] and Rahmani et al. [17], using a variational approach.

Consider a flat Lorentz-invariant Minkowski space-time, $x^{\alpha }=\left(ct,x\right)$, assuming the invariance of the element $ds^{2}=c^{2}dt^{2}-{\left|dx\right|}^{2}=\eta_{\alpha \beta }dx^{\alpha }dx^{\beta }$, where $\eta_{\alpha \beta }$ is the Minkowski metric, i.e., $\eta_{\alpha \beta }=diag\left(1,-1,-1,-1\right)$. Consequently, defining the covariant and contravariant gradient operators [18],

(39) $$\partial_{\alpha }=\frac{\partial }{\partial x^{\alpha }}=\left(\frac{1}{c}\frac{\partial }{\partial t},\nabla \right);\;\partial^{\alpha }=\frac{\partial }{\partial x_{\alpha }}=\left(\frac{1}{c}\frac{\partial }{\partial t},-\nabla \right)=\eta^{\alpha \beta }\partial_{\beta }.$$

shows that the d’Alambertian corresponds indeed to a four-dimensional Laplacian, i.e.,

(40) $$□=\partial^{\alpha }\partial_{\alpha }=\eta_{\alpha \beta }\partial^{\alpha }\partial^{\beta }=\frac{1}{c^{2}}\frac{\partial^{2}}{\partial t^{2}}-\nabla^{2}.$$

Here, the relative time *t* is related to the proper time τ (i.e., measured in the co-moving reference frame) as dt=γdτ, where $\gamma ={\left(1-\beta^{2}\right)}^{-1/2}$, with β=|v|/c and v=dr/dt is the 3D velocity. Note that $u_{\alpha }=dx_{\alpha }/dt$ should not be confused with the 4-velocity $U_{\alpha }=dx_{\alpha }/d\tau =\gamma u_{\alpha }=\gamma \left(c,v\right)$, with $U_{\alpha }U^{\alpha }=c^{2}$.

Finally, proceeding as in Equations (19)–(25), we find the chemical potential to be $\mu =\mu_{th}+\mu_{nl}$, where $\mu_{th}$ is unchanged from the non-relativistic case, while

(41) $$\mu_{nl}=V_{Q}=-\partial_{\alpha }\frac{\partial \Delta f_{nl}}{\partial \partial_{\alpha }\rho }=\frac{\hbar^{2}}{2}\frac{□\sqrt{\rho }}{\sqrt{\rho }},$$

which generalizes Equation (25).

Now we can write the governing equations. First, the continuity Equation (3) in covariant form reads

(42) $$\partial_{\alpha }J^{\alpha }=0,\;or\;\eta_{\alpha \beta }\partial^{\alpha }J^{\beta }=0,$$

where $J^{\alpha }=\left(\rho c,\rho v\right)$ is the 4-flux (that is, the 4-momentum per unit volume). Additionally, $J^{\alpha }=\rho u^{\alpha }$, where $u^{\alpha }=\left(c,v\right)$ is the velocity (that is, the 4-momentum per unit mass).

As for the momentum balance equation, the equation of motion for an ideal fluid, that is free of any dissipative effects, can be obtained assuming that Equations (4) and (31) must be reduced in the non-relativistic limit, obtaining, refs. [18,19,20]

(43) $$\partial_{\alpha }T^{\alpha \beta }=G^{\beta },$$

where

(44) $$T^{\alpha \beta }=-P\eta^{\alpha \beta }+\left(\frac{P}{c^{2}}+\rho \right)U^{\alpha }U^{\beta },$$

with $U^{0}=\gamma c$, and U=γv is the energy-momentum 4-tensor, while

(45) $$G^{\alpha }=-\partial^{\alpha }\left[\left(V+V_{Q}\right)\rho \right]$$

is the force density. Finally, the following governing equation is obtained:

(46) $$-\partial^{\beta }h+\partial_{\alpha }\left[\left(\frac{P}{c^{2}}+\rho \right)U^{\alpha }U^{\beta }\right]=0,$$

where *h* is the enthalpy per unit volume, i.e.,

(47) $$h=-\rho^{2}\frac{\partial \mu }{\partial \rho }=P+\left(V+V_{Q}\right)\rho .$$

Thus, in agreement with Tavernelli [21], we see that particles move deterministically along geodesic lines in a curved space whose curvature depends nonlocally on Bohm’s quantum potential.

## 4. Comments and Conclusions

In this work, we propose a covariant formulation of quantum mechanics. For this, we start from a particular formulation of quantum mechanics, that is the Madelung equation, and show that it can be derived from classical mechanics assuming that the energy of a system depends on its density through a non-local functional. At the end, we find Newton’s equation of motion where the energy of the system contains an extra term, namely, the so-called Bohm’s quantum potential. Clearly, as any density change at a point determines an instantaneous energy variation at another point, this non-local approach has built in the same limitation as the Schrödinger equation, that is, it assumes an infinite propagation velocity of any density perturbation, and therefore it cannot be Lorentz-invariant (see discussion in [14]). So, first we generalize the non-local energy-density functional dependence, introducing a retarded potential, and consequently derive a covariant, Lorentz-invariant extension of Bohm’s quantum potential. Then, when this extra potential is introduced into the relativistic equation of a perfect fluid, we obtain the governing Equation (46) which, we believe, is both Lorentz-invariant and quantum-compatible.

What remains to be seen, obviously, is the nature of the non-local interactions that we have introduced, whose coarse-grained approximation would constitute quantum mechanics (see discussion in Smolin [22]).

## Data Availability

Not applicable.
